# The impact of reduced intraoperative mean arterial pressure on postoperative hemorrhage in bariatric surgery

**DOI:** 10.1007/s00464-025-11841-y

**Published:** 2025-06-16

**Authors:** Arturo Estrada, Yeisson Rivero-Moreno, Jasson Xia, Diego Zamata-Ovalle, Karen Velez, Jorge Humberto Rodriguez-Quintero, Jenny Choi, Erin Moran-Atkin, Diego Camacho

**Affiliations:** Department of Surgery, Montefiore Medical Center, 3400 Bainbridge Avenue, 4 Th Floor, Bronx, NY 10467 USA

**Keywords:** Postoperative hemorrhage, Bariatric surgery, Mean arterial pressure, Roux-en-Y gastric bypass, Sleeve gastrectomy, Intraoperative hypotension

## Abstract

**Background:**

Postoperative hemorrhage (POH) is a life-threatening complication, occurring in 1.3–1.7% of bariatric surgeries and still constitutes a recognized challenge. This study examined the effect of intraoperative mean arterial pressure (MAP) on the development of POH.

**Methods:**

A retrospective observational study with a case–control design was conducted on adult patients who underwent bariatric surgery between 2015 and 2023 at a high-volume academic center. Intraoperative MAP (including MAP in the last 10 and 30 min) was collected in patients who developed POH. Cases were matched with controls by sex, gender, type of procedure, and ASA classification.

**Results:**

From 204 participants, 102 patients with POH were matched with 102 controls. The most common procedure performed was Roux-en-Y gastric bypass (*n* = 98, 48%), followed by sleeve gastrectomy (*n* = 77, 37.7%). Patients with POH had statistically significant lower intraoperative MAP during the last 10 min (92.41 ± 14.25 vs 97.44 ± 14.64, *p* = 0.014) and 30 min (87.93 ± 12.32 vs 91.93 ± 11.26, p = 0.016) of surgery compared to controls. An intraoperative MAP lower than 90 mmHg in the last 10 min (OR = 2.067, 95% CI = 1.156–3.695), 30 min (OR = 2.231, 95% CI = 1.27–3.919), and whole procedure (OR = 1.834, 95% CI = 1.024–3.285) was associated with increased risk of POH. No significant differences were found in comorbidities, smoking, preoperative laboratory results, history of antiplatelet therapy or anticoagulation use, and operative time between the two groups.

**Conclusion:**

Our study demonstrates that patients with POH had lower intraoperative MAP during the last 10 and 30 min of surgery. An intraoperative MAP < 90 mmHg was identified as a risk factor for developing POH.

The obesity pandemic poses significant health challenges in the twenty-first century with metabolic and bariatric surgery (MBS) evolving into a potent tool not only for achieving weight loss but also for inducing the remission of different obesity-associated diseases [1]. In the United States alone, nearly 280,000 bariatric procedures were performed in 2022, according to the American Society for Metabolic and Bariatric Surgery [2].

Currently, bariatric surgery is performed safely at certified centers but still carries the risk of serious complications. The risk of death associated with bariatric surgery is approximately 0.1%, and the overall likelihood of major complications is about 4% [3]. Some early complications include leaks, bleeding, and venous thromboembolic events. Late complications can involve dumping syndrome, marginal ulcers, blind loop syndrome, vitamin deficiencies, and stomal stenosis [4].

The most frequently reported complication is POH, which occurs in 0.4–4.4% after gastric bypass and in 0.4–3.4% after sleeve gastrectomy (SG). The severity of POH can range from self-limiting to severe and life-threatening situations. For this reason, major POH is the leading cause of increased postoperative morbidity and extended hospital stay in most studies [5].

Various factors have been identified as risks for POH following bariatric surgery, including male gender, advanced age, cardiovascular diseases, and revisional surgery, among others [6, 7]. Some studies have suggested a possible association between intraoperative MAP and POH after MBS, but the results have been contradictory [8, 9]. The exact relationship and direction of the effect of intraoperative MAP on POH remain to be determined.

Understanding the risk factors linked to POH is crucial for optimizing modifiable risks before surgery and for individual risk stratification, which can lead to more vigilant monitoring during the early postoperative period. This study aimed to evaluate what is the exact impact of intraoperative MAP in the development of POH in patients after MBS.

## Materials and methods

A retrospective observational study with a case–control design was conducted on patients who underwent bariatric surgery at a high-volume academic center in New York, which performs over a thousand bariatric cases per year.

The patient population comprised adults (aged 21 and older) who underwent bariatric procedures, including SG, Roux-en-Y gastric bypass (RYGB), conversion from SG to RYGB, and RYGB revision, from January 2015 to December 2023. Cases were defined as patients who experienced POH within the first 30 days after MBS. In this study, POH was defined as a decrease in hemoglobin of ≥ 2 g/dL over 48 h, the requirement for blood transfusion, hematemesis, melena, hematochezia, and/or radiological evidence of hemorrhage (such as free fluid on ultrasound or computed tomography) within the subsequent 30 days postoperatively. Controls, or patients without POH, were randomly selected from the database of all bariatric patients in the center, using the tools of the statistical software (SPSS) to match the initial cases based on the following criteria: age, gender, type of surgical procedure, and American Society of Anesthesiologists (ASA) classification. The final sample of patients was determined by those who experienced POH (*n* = 102) (convenience sampling) and the controls (*n* = 102) for these patients at a 1:1 ratio.

All operations were performed robotically or laparoscopically by the same team of bariatric surgeons. Preoperatively, all patients received a prophylactic dose of subcutaneous heparin at 5000 IU. The intra-abdominal pneumoperitoneum pressure was maintained at 15 mmHg for the entire operation, with no routine adjustments performed. For laparoscopic procedures, a Medtronic EndoGIA stapler (with a 45–60 mm black or purple load) was utilized to create the sleeve or pouch. In robotic cases, a robotic Intuitive SureForm stapler (with a 60 mm green or blue load) was employed for the same purpose. In both primary RYGB and sleeve-to-RYGB conversion cases, the technique for the gastrojejunostomy varied between circular stapler (25 mm EEA circular stapler), linear stapler (EndoGIA 45 mm purple load, and the common enterotomy closed with 2–0 Polyglactin 910), or hand-sewn (two-layer hand-sewn with 2–0 Polyglactin 910), depending on the surgeon’s preference. After all procedures, a leak test was performed using air insufflation through a 34-French bougie; routine endoscopy was not performed. After all procedures, sites of gastrectomies and intestinal anastomosis were covered with VISTASEAL™ Fibrin Sealant. As per our institutional protocol, all bariatric patients are discharged with prophylactic-dose enoxaparin for 28 days.

Data on age, sex, type of procedure, preoperative BMI, smoking history, comorbidities, use of antiplatelet therapy (APT), use of anticoagulation (AC), preoperative laboratory results (hemoglobin, hematocrit, albumin, and International Normalized Ratio), postoperative hemoglobin, transfusions, hemorrhage manifestations, operative time (OT), intraoperative MAP, and American Society of Anesthesiologists (ASA) classification were extracted from the hospital’s database. These data were used to calculate the mean MAP for the entire procedure, as well as the mean MAP for the last 10 and 30 min.

Quantitative variables were expressed as median and standard deviation (SD), and qualitative variables as proportions. The Chi-square and Mid-P exact test were used for categorical variables, and the Student’s t test was used for quantitative variables. Subgroup analysis was performed to evaluate possible cofounders. Odds ratios with a 95% confidence interval (CI) were used to evaluate the relationship between MAP < 70 mmhg, < 80 mmhg, < 90mmhg, and < 100mmhg and the occurrence of postoperative hemorrhage. The statistical software IBM SPSS Statistics for Windows, Version 29.0.2.0 (Armonk, NY: IBM Corp) was used to conduct statistical tests to assess for statistical significance (p < 0.05).

The procedures performed on the studied patients, as well as the management of the data, were in accordance with the ethical standards of the institutional research committee (IRB #2023–15074) and with the 1964 Helsinki Declaration and its later amendments or comparable ethical standards. No informed consent was required for the development of this project. This study was conducted following the guidelines and recommendations of the STROBE guidelines [10].

## Results

Data from 204 patients who underwent bariatric surgery were analyzed. The patients were divided into two groups of 102 each based on the occurrence of POH. A total of 87.7% (*n* = 179) of patients were female, with a mean age of 45.7 ± 11.4 years and a mean BMI of 43.86 ± 7.94, with no statistically significant difference between groups.

In our patient sample, 35.3% had diabetes mellitus (DM), 36.6% had obstructive sleep apnea (OSA), 35.3% had gastroesophageal reflux disease (GERD), 48.5% had hypertension (HTN), 32.4% had hyperlipidemia (HLD), and 13.7% had a history of smoking. When comparing the two groups, the POH group had higher frequencies; however, no statistically significant difference was found. Preoperatively, APT, including acetylsalicylic acid and clopidogrel, was documented in 18.1% of our patients, while only 1% received AC therapy.

The mean preoperative albumin was 4.2 ± 0.31 gr/dl, the international normalized ratio (INR) was 0.98 ± 0.07, preoperative hematocrit was 40.42 ± 3.86%, and preoperative hemoglobin was 12.82 ± 1.46 gr/dl, with no significant difference observed between groups.

The most prevalent ASA classification was class III, accounting for 80.9% (n = 165) of patients, followed by class II at 18.1% (*n* = 37) and class IV at 1% (*n* = 2). Detailed characteristics and differences between the two patient groups are presented in Table 1.

**Table 1:** Characteristics of patients undergoing bariatric surgery based on the occurrence of POH

	Overall (*n* = 204)	Cases (*n* = 102)	Control (*n* = 102)	*P*-value
*Characteristics*^a^				
BMI (kg/m^2^)	43.86 ± 7.94	44.25 ± 8.62	43.75 ± 7.22	0.485^c^
Sex – Female	179 (87.7)	88 (86.3)	91 (89.2)	0.522^b^
Age	45.7 ± 11.4	45.06 ± 11.33	46.38 ± 11.50	0.410^c^
Smoker	28 (13.7)	17 (16.7)	11 (10.8)	0.222^b^
DM	72 (35.3)	39 (38.2)	33 (32.4)	0.379^b^
OSA	74 (36.6)	34 (33.3)	40 (39.2)	0.382^b^
GERD	72 (35.3)	39 (38.2)	33 (32.4)	0.379^b^
Hypertension	99 (48.5)	56 (54.9)	43 (42.2	0.069^b^
Hyperlipidemia	66 (32.4)	38 (37.3)	28 (27.5)	0.134^b^
Use of APT	37 (18.1)	18 (17.6)	19 (18.6)	0.855^b^
Use of AC	2 (1)	0	2 (2)	0.248^d^
Pre-op Albumin (gr/dl)	4.2 ± 0.31	4.24 ± 0.31	4.16 ± 0.3	0.058^c^
Pre-op INR	0.98 ± 0.07	0.98 ± 0.076	0.99 ± 0.060	0.688^c^
Pre-op Hct (%)	40.42 ± 3.86	40.63 ± 3.98	40.22 ± 3.74	0.455^c^
Pre-op Hb (gr/dl)	12.82 ± 1.46	12.82 ± 1.48	12.79 ± 1.41	0.912^c^
ASA II	37 (18.1)	17 (16.7)	20 (19.6)	0.585^b^
ASA III	165 (80.9)	84 (82.4)	81 (79.4)	0.593^b^
ASA IV	2 (1)	1 (1)	1 (1)	> 0.999^d^

*BMI* body mass index, *DM* diabetes mellitus, *OSA* obstructive sleep apnea, *GERD* gastroesophageal disease, *APT* antiplatelet therapy, *AC* anticoagulation, *INR* international normalized ratio, *OT* operative time, *ASA* American Society of Anesthesiologists

^a^Continuous data are shown as the mean ± standard deviation and categorical data as number and (percentage)

^b^Chi-square

^c^T-Student

^d^Mid-P exact

Bold values are statistically significant

The most common procedure performed was RYGB, accounting for 48% (*n* = 98) of cases, followed by SG at 37.7% (*n* = 77), conversion from SG to RYGB at 8.3% (*n* = 17), and RYGB revision at 5.9% (*n* = 12). We found that patients in the POH group underwent conversion from SG to RYGB less frequently compared to the control group (2% vs. 14.7%, *p* > 0.001).

Upon evaluating the MAP, it was found to be lower in the case group (POH) compared to the control group during the last 30 min (87.93 ± 12.32 vs 91.93 ± 11.26, *p* = 0.016) and the last 10 min (92.41 ± 14.25 v 97.44 ± 14.64, *p* = 0.016) of the procedure. However, no difference was observed when evaluating the mean MAP for the entire procedure. (Table 2).

**Table 2:** Intraoperative and post-operative characteristics of patients undergoing bariatric surgery based on the occurrence of POH

	Overall (*n* = 204)	Cases (*n* = 102)	Control (*n* = 102)	*P*-value
*Characteristics*^a^				
Type of procedure				
SG, n (%)	77 (37.7)	32 (31.4)	45 (44.1)	0.064^b^
RYGB, n (%)	98 (48)	49 (48)	49 (48)	> 0.999^b^
CS^d^	38 (38.8)	20 (40.8)	18 (36.7)	0.678^b^
LS^d^	29 (29.6)	13 (26.5)	16 (32.7)	0.506^b^
HS^d^	31 (31.6)	16 (32.7)	15 (30.6)	0.828^b^
Conversion SG-RYGB, n (%)	17 (14.7)	15 (14.7)	2 (2)	** < 0.001**^b^
CS^d^	8 (47)	7 (46.6)	1 (50)	0.929^b^
LS^d^	3 (17.6)	3 (20)	0 (0)	–
HS^d^	6 (35.3)	5 (33.3)	1 (50)	0.787^b^
RYGB Revision, n (%)	12 (5.9)	6 (5.9)	6 (5.9)	> 0.999^b^
OT (min), mean ± SD	96.7 ± 44.98	98.16 ± 37	95.21 ± 51.89	0.637^c^
Intraoperative MAP				
MAP last 10 min, mean ± SD	94.92 ± 14.63	92.41 ± 14.25	97.44 ± 14.64	**0.014**^c^
MAP last 30 min, mean ± SD	89.93 ± 11.94	87.93 ± 12.32	91.93 ± 11.26	**0.016**^c^
MAP whole procedure, mean ± SD	86.15 ± 10.1	85.03 ± 11.11	87.27 ± 8.89	0.113^c^
Post-op Hb (gr/dl), mean ± SD	11.02 ± 1.94	9.81 ± 1.86	12.21 ± 1.44	** < 0.001**^c^

*SG* sleeve gastrectomy, *RYGB* Roux-en-Y gastric bypass, *OT* operative time, *MAP* mean arterial pressure (mmhg), *CS* Circular stapler, *LS* linear stapler, *HS* hand sewn

^a^Continuous data are shown as the mean ± standard deviation and categorical data as number and (percentage)

^b^Chi-square

^c^T-Student

^d^Specific technique used for the gastrojejunal anastomosis

Bold values are statistically significant

Patients in the POH group had lower hemoglobin levels 24 h postoperatively compared to the control group (9.8 ± 1.86 vs. 12.2 ± 1.4, *p* < 0.001). Additionally, the mean lowest postoperative hemoglobin level in the POH group was 7.42 ± 1.1, primarily recorded on postoperative day 2 in 30% of patients.

Further analysis was conducted to evaluate the risk of POH based on intraoperative MAP. Assessing various MAP thresholds at different times during the procedure, it was found that an intraoperative MAP below 90 mmHg during the last 10 min (OR = 2.067, 95% CI = 1.156–3.695), the last 30 min (OR = 2.231, 95% CI = 1.27–3.919), and throughout the entire procedure (OR = 1.834, 95% CI = 1.024–3.285) was associated with an increased risk of POH (see Table 3).

**Table 3:** Association between intraoperative MAP and risk of postoperative bleeding

Intraoperative MAP	Cases (*n* = 102)	Controls (*n* = 102)	OR (95% CI)
< 70 mmhg			
Last 10 min	4 (3.9)	1 (1)	4.122 (0.452–37.536)
Last 30 min	8 (7.8)	1 (1)	**8.595 (1.054–70.036)**
Whole procedure	6.8 (6.8)	2 (2)	3.684 (0.746–18.181)
< 80 mmhg			
Last 10 min	19 (18.6)	11 (10.8)	1.893 (0.85–4.214)
Last 30 min	27 (26.5)	17 (16.7)	1.8 (0.91–3.558)
Whole procedure	35 (34.3)	25 (24.5)	1.608 (0.875–2.957)
< 90 mmhg			
Last 10 min	46 (45.1)	29 (28.4)	**2.067 (1.156–3.695)**
Last 30 min	66 (64.7)	46 (45.1)	**2.231 (1.27–3.919)**
Whole procedure	73 (71.6)	59 (57.8)	**1.834 (1.024–3.285)**
< 100 mmhg			
Last 10 min	75 (73.5)	62 (60.8)	1.792 (0.99–3.242)
Last 30 min	84 (82.4)	80 (78.4)	1.283 (0.641–2.569)
Whole procedure	93 (91.2)	94 (92.2)	0.879 (0.325–2.377)

*MAP* mean arterial pressure, *CI* confidence interval

Bold values are statistically significant

A subgroup analysis was conducted based on the type of bariatric procedure for conversion SG-RYGB vs other Type, with no statistically significant difference found in terms of MAP between the case and control group in the different. Details are presented in Table 4.

**Table 4:** Subgroup analysis of intraoperative MAP base on type of bariatric procedure

	Conversion SG-RYGB (*n* = 17)			No conversion SG-RYGB (*n* = 187)		
Intraoperative MAP	Cases (*n* = 15)	Control (*n* = 2)	*P*-value	Cases (*n* = 87)	Control (*n* = 100)	*P*-value
MAP last 10 min, mean ± SD	88.2 ± 10.9	104 ± 10	0.064	93.1 ± 14.7	97.3 ± 14.7	0.055^a^
MAP last 30 min, mean ± SD	82.2 ± 11.3	95.9 ± 9.2	0.127	88.9 ± 12.3	91.8 ± 11.3	0.091^a^
MAP whole procedure, mean ± SD	78 ± 10.1	93.8 ± 3.9	0.053	86.2 ± 10.8	87.1 ± 8.9	0.533^a^

*SG* sleeve gastrectomy, *RYGB* Roux-en-Y gastric bypass, *MAP* mean arterial pressure (mmhg)

^a^T-Student

## Discussion

Postoperative hemorrhage is among the most common early complications, as reported in the literature, and can occur both intraluminally and intra-abdominally (e.g., from the staple line, omentum, or port sites) [11]. Early postoperative bleeding is correlated with prolonged hospitalization, serious complications, reoperation, and increased mortality.

We present a large single-institution retrospective case–control study that demonstrates a significant association between intraoperative MAP and the development of POH. Multiple studies have examined intraoperative blood pressure regulation as a key factor in the occurrence of this complication [9, 12]. In our study, intraoperative MAP in the POH group was found to be lower during both the last 30 min and the last 10 min of the procedures.

Many methods have been described to decrease the rate of POH, including oversewing staple lines, bovine or polymer membranes, and fibrin glue [13]. Of the described methods, intraoperative blood pressure control is an uncomplicated intervention that has both minimal potential complications and minimal effect on operative time and costs. Recent studies have associated intraoperative MAP with POH after MBS; however, results have been contradictory. Sroka et. al. initially studied the effects of blood pressure elevation to 140 mmHg in combination with biologic glue or oversewing staple lines in laparoscopic sleeve gastrectomies and found that the combination of oversewing staple lines with blood pressure elevation minimized hemorrhagic complications [11]. Subsequently, Ying et. al. in 2019 explored the effect of intraoperative blood pressure alone on POH and found that patients with POH had a lower MAP in the last 15–25 min of each case. However, this study only had eight patients with POH [14]. In 2021, Janik et. al. conducted a slightly larger retrospective case–control study with 12 matched pairs and found that POH was associated with significantly increased MAP 5 min prior to the completion of the case for laparoscopic sleeve gastrectomy. These results were contrary to prior studies and showed that further research needed to be conducted [15].

Two newer studies have now made a stronger case for increasing intraoperative MAP as a preventative measure to decrease rates of POH. In 2022, Pavone et. al. created a protocol in which they increased intraoperative MAP by 30% and decreased CO2 pneumoperitoneum pressure to 8 mmHg in the last 15 min of laparoscopic sleeve gastrectomies. They found that with this combination of adjusted MAP and decreased intra-abdominal pressure at the end of surgery, POH decreased significantly [16]. Fink et. al. then conducted a retrospective observational analysis in 2024 in which cases before 2015 were used as the control group, and cases performed after 2015 included an artificial elevation of intraoperative systolic blood pressure to 150 mmHg. They found a significant decrease in POH rate in the later cohort, although this study does not use intraoperative MAP and has significant confounding factors [9].

Compared to prior literature, our study focused on intraoperative MAP, specifically the last 10 and 30 min. Our study contains one of the largest cohorts available in the literature for POH after bariatric surgery and contributes to the current data suggesting that there exists a critical window of time in which potential bleeding sources may be identified at the end of a case. When the intraoperative blood pressure is low during this critical window of time, areas of potential bleeding may remain inactive and hinder the ability of a surgeon to ensure adequate hemostasis. It is possible that after blood pressures return to normal levels after anesthesia, these areas begin to bleed and thus lead to POH.

This study is the first to identify a clear target for blood pressure monitoring intraoperatively. Although two prior studies have targeted a 30% increase in MAP or a systolic blood pressure of 150 mmHg in their interventions, we are able to establish that a significant increase in risk is associated with an intraoperative MAP < 90 mmHg. Additionally, recent guidelines from the Enhanced Recovery After Surgery (ERAS) Society for Bariatric Surgery strongly recommend that intraoperative goals should include maintaining euvolemia and normotension, as well as preventing acute anemia from blood loss, to ensure adequate tissue oxygenation at the anastomosis site [12]. The observed findings should encourage more precise and strict control of intraoperative MAP. Increasing intraoperative blood pressure to assess for bleeding is a common surgical maneuver, particularly following major procedures or when hemostasis remains uncertain. These include reducing the depth of anesthesia or administering short-acting vasopressor boluses such as phenylephrine or ephedrine. [11] This insight not only aids in guiding intraoperative management to potentially reduce the incidence of POH, but also underscores the importance of conducting future studies to evaluate whether targeted interventions to maintain or adjust MAP during bariatric surgery could effectively reduce POH rates. These findings should serve as a foundation for prospective research and randomized clinical trials aimed at shaping new evidence-based guidelines for intraoperative blood pressure management in bariatric patients.

The main limitation of this study is the single-institution, case–control design. Although conducted at a high-volume center with the same team of surgeons, this study collected patient data from 2015 to 2023 in order to achieve its comparatively large and homogenous sample size. Additionally, the nature of the retrospective design conveys limited control over the nature and quality of the predictor variables. As a result, confounding factors may exist, such as variations in surgical technique and skill across the eight years.

For patients who developed POH, intraoperative MAP was lower during both the last 30 min and the last 10 min of the procedure. Additionally, a mean intraoperative MAP below 90 mmHg was associated with an increased risk of POH. Future large-scale and prospective studies will be required to determine the optimal intraoperative blood pressure goals to minimize POH.
